# Acute Stroke Patients on Daily Anticoagulant or Antiplatelet Therapy Receiving Thrombolysis or Thrombectomy

**DOI:** 10.7759/cureus.106657

**Published:** 2026-04-08

**Authors:** Scott M Alter, Salil Phadnis, Zuheir Mirza, Joshua J Solano, Richard D Shih, Patrick G Hughes, Lisa M Clayton

**Affiliations:** 1 Department of Emergency Medicine, Florida Atlantic University Charles E. Schmidt College of Medicine, Boca Raton, USA; 2 Department of Emergency Medicine, Washington University in Saint Louis School of Medicine, St. Louis, USA; 3 Department of Emergency Medicine, University of Utah School of Medicine, Salt Lake City, USA

**Keywords:** direct oral anticoagulation drugs, intracranial hemorrhage (ich), post-stroke recovery, stroke guidelines, tpa treatment

## Abstract

Objective

The use of anticoagulant and antiplatelet medication has traditionally been cited as a contraindication for acute ischemic stroke treatments. However, the 2026 American Heart Association (AHA)/American Stroke Association (ASA) Guidelines have updated these standards, designating tenecteplase (TNK) as a preferred thrombolytic agent and identifying the benefit-to-risk ratio for thrombolysis in patients with recent anticoagulant exposure as uncertain. Conversely, mechanical thrombectomy is increasingly utilized regardless of antithrombotic status. This study examines the difference in post-intervention intracranial hemorrhage (ICH) and modified Rankin score (mRS) between patients receiving comprehensive stroke treatment who were taking these medications versus patients who were not on antithrombotic pharmacotherapy.

Methods

This study was a retrospective chart review. Inclusion criteria consisted of adult patients who were evaluated in the emergency department for stroke and received either thrombolysis (tissue plasminogen activator (tPA) or TNK) or procedural neurointervention between March 2018 and May 2020. Data collected included patient demographics, pre-stroke antiplatelet use, anticoagulant use, post-intervention mRS, and incidence of ICH. ICH and mRS outcomes were compared between patients based on their antithrombotic use for each of the neurointervention groups.

Results

A total of 251 patients met the inclusion criteria. Patients taking antiplatelets who received both thrombolysis and thrombectomy had significantly higher mRS scores compared to patients not taking these agents. Patients taking antiplatelets who received thrombolysis alone tended to have higher mRS but failed to reach significance. Patients taking only anticoagulants showed no difference in mRS for any intervention. Patients taking both anticoagulants and antiplatelets had a higher mRS than those not taking either medication. Crucially, there were no significant differences in the rate of ICH after any intervention regardless of medication usage. There was also no difference in any outcomes between patients on single versus dual antiplatelets.

Conclusion

Our results suggest that patients on antiplatelet and anticoagulant medication may be able to safely receive procedural neurointervention and thrombolysis for acute ischemic stroke without an increased risk of ICH. While functional outcomes (mRS) were higher in medicated groups, these results likely reflect underlying comorbidities rather than procedural complications. These hypothesis-generating findings align with the evolving 2026 clinical landscape and underscore the need for prospective studies to resolve current clinical uncertainties regarding antithrombotic status and reperfusion therapy.

## Introduction

Background

Annual incidence of stroke in the United States (US) is approximately 800,000, with 87% of these being ischemic strokes, most with thrombotic or embolic etiologies. Approximately 600,000 are first-time strokes, and 200,000 are recurrent strokes [1]. Patients experiencing their first stroke often already have multiple comorbidities which require ongoing antiplatelet and anticoagulant use.

Comprehensive care of the ischemic stroke patient consists of chemical and mechanical thrombolysis. The administration of thrombolytics was traditionally based on the original National Institute of Neurological Disorders and Stroke (NINDS) I/II and European Cooperative Acute Stroke Study (ECASS) III trials, which utilized alteplase and established oral anticoagulant use as a clear exclusion criterion [2,3]. However, the 2026 American Heart Association (AHA)/American Stroke Association (ASA) Guidelines now identify tenecteplase (TNK) as a preferred thrombolytic agent (Class 1) and have modified the approach to pre-stroke antithrombotics [4]. While mechanical thrombectomy (endovascular thrombectomy (EVT)) has become the standard of care for large vessel occlusions (LVO), the clinical guidelines for its use differ significantly from chemical thrombolysis when a patient is anticoagulated. The use of procedural neurointervention varies depending on institutional guidelines and individual neurointerventionalists.

Importance

Both chemical and mechanical neurointervention have classically been contraindicated in patients receiving antithrombotic medications due to concern for increasing the likelihood of post-intervention intracranial hemorrhage [5]. The 2026 guidelines highlight a growing divergence in these recommendations: while mechanical thrombectomy is generally considered safe and recommended for eligible patients regardless of anticoagulant status, the safety of chemical thrombolysis with TNK or alteplase in patients with recent direct oral anticoagulant (DOAC) exposure remains uncertain [4]. The risk of hemorrhagic conversion already increases with neurointervention due to reperfusion injury and thrombus fragmentation with distal migration and damage to the vascular bed, which has the potential to significantly increase morbidity and mortality [6]. Physicians evaluating a patient with acute ischemic stroke must carefully balance the risk of hemorrhage with the benefit of reperfusion. The prevailing concern is that while mechanical interventions bypass systemic lytic risks, the use of chemical thrombolytics in anticoagulated patients may carry a risk-to-benefit ratio that is not yet fully established. This study aims to determine if these extra precautions unnecessarily prevent patients from benefiting from highly effective stroke treatments.

Goals of this investigation

This study examines pre-stroke antiplatelet and anticoagulant medication use in patients who received either tissue plasminogen activator (tPA) or procedural neurointervention. Specific outcome measures include incidence of post-intervention intracranial hemorrhage (ICH) and modified Rankin score (mRS) upon hospital discharge [7].

## Materials and methods

Study design and setting

This study was a retrospective chart review performed at a comprehensive stroke center in South Florida. The study was approved by the hospital’s research committee and the institutional review board of its affiliated university (Florida Atlantic University Health Sciences IRB, Boca Raton, USA; approval number 1635774-1; dated August 10, 2020). At the study institution, patients who present with new neurologic deficits that began within 24 hours trigger a “stroke alert”. This mobilizes the stroke team, consisting of the emergency physician and a neurologist, who jointly stabilize the patient and perform a rapid assessment and NIH stroke scale [2]. In addition, a neurointerventionalist is immediately consulted. All patients receive non-contrast CT head imaging. CT head/neck angiogram and CT brain perfusion studies are also completed unless there is a known contrast allergy, evidence of intracranial hemorrhage on non-contrast CT, or the patient is deemed not to be an interventional candidate based on mRS score. A board-certified radiologist, not involved with the research study, reviews and provides a report on all imaging studies. Intravenous tPA is administered for strokes that begin within 4.5 hours. History of concurrent hemorrhage, anticoagulant use, and alternative etiologies of the presenting symptoms are obtained by the treating physician. Warfarin use with an international normalized ratio (INR) greater than 1.7 or direct oral anticoagulant (DOAC) use are considered contraindications. If a large vessel occlusion (LVO) is present, the on-call neurointensivist evaluates the patient for thrombectomy. Following treatment, patients have repeat imaging of the brain, with non-contrast CT, MRI, or both. All stroke alert patients are entered into the hospital’s stroke registry. The stroke registry is a hospital-wide database that tracks and measures performance and outcomes in patients receiving reperfusion therapy for acute ischemic stroke. It is maintained by the hospital stroke committee comprising physicians, nurses, and administrators and follows guidelines established by the statewide Florida Stroke Registry.

Patient selection

Consecutive patients listed in the study hospital’s stroke registry between March 2018 and May 2020 were screened for eligibility by a physician investigator. Those who had tPA administration or thrombectomy were included in this research study. There were no specific exclusion criteria.

Measurements

After meeting screening criteria, patient medical record numbers were imported from the hospital’s stroke registry into REDCap (Vanderbilt University, Nashville, Tennessee, USA), a data management platform. A standardized instrument was utilized for consistent data entry with real-time parameter validation performed. Data was abstracted by one of the physician investigators, who regularly met with another physician investigator to review the data and resolve disputes.

Variables collected included age, gender, initial ED vital signs, pre-intervention National Institutes of Health Stroke Scale (NIHSS), antiplatelet therapy use, and anticoagulation therapy use. Age and gender were obtained from patient-provided information to the registration clerk. Initial ED vital signs were obtained from the nursing triage note. Antiplatelet and anticoagulant medication use was determined from patient self-reporting, chart review from previous hospital visits in the Electronic Medical Record (EMR), and pharmacy history of prescriptions imported into the EMR. Type of initial ED CT head imaging performed was recorded as CT non-contrast, CT angiography, and CT perfusion. CT non-contrast results were categorized as normal, acute infarct, subacute infarct, old infarct, acute ICH, or chronic ICH. CT angiography results were categorized as normal or large vessel occlusion. CT perfusion results were categorized as normal, core, or penumbra. Patient diagnoses based on physician notes were recorded as ischemic stroke, hemorrhagic stroke, subarachnoid hemorrhage, or not a stroke. For each patient, the type of treatment received was recorded: tPA, neurointervention with thrombectomy, or both. Any neuroimaging that occurred for three months following neurointervention was reviewed to identify incidence of ICH. mRS was obtained upon review of documentation from physical therapy, occupational therapy, and physical medicine and rehabilitation specialists. Post-intervention intracranial hemorrhage was defined as any new area of hyperdensity on follow-up computed tomography (CT) or magnetic resonance imaging (MRI) compared to baseline. While our primary safety endpoint included all radiographic ICH, we did not specifically categorize events as symptomatic (sICH) versus asymptomatic, as the clinical documentation of neurological decline specifically attributable to the hemorrhage was not consistently available in this retrospective cohort.

Outcomes

The primary outcomes were mRS and incidence of ICH following each type of neurointervention. ICH was diagnosed on CT head or MRI brain when new hemorrhage was visualized following neurointervention as compared to imaging taken prior to neurointervention.

Analysis

Several comparative analyses were performed based on antithrombotic medication use at the time of stroke onset: (1) antiplatelet alone (antiplatelet group) versus none (no antithrombotic group: anticoagulant and antiplatelet agents excluded), (2) anticoagulant alone (anticoagulant group) versus no antithrombotic group, (3) combined antiplatelet and anticoagulant (combined group) versus no antithrombotic group, and (4) single antiplatelet versus dual antiplatelet (anticoagulant agents excluded). Patients were grouped based on neurointervention: tPA, thrombectomy, or both. Chi-squared tests were used to compare incidence of ICH, while Mann-Whitney tests were used to determine the differences in mRS. Mann-Whitney tests were also used to determine the relationship between initial NIHSS and patients taking antiplatelet or anticoagulant therapies.

A significant portion of patients (n=130) lacked a documented initial NIHSS score in the electronic medical record. To maintain the statistical power of the study, these individuals were excluded only from analyses specifically involving NIHSS, while remaining in the primary cohorts for the analysis of ICH incidence and discharge mRS outcomes. Statistical analyses were performed using SPSS Statistics version 29.0 (IBM Corporation, Armonk, NY, USA).

## Results

Background characteristics

Between March 2018 and May 2020, 663 patients had stroke activations. Of these, 251 patients received neurointervention: 140 (55.8%) received tPA only, 77 (30.7%) had thrombectomy only, and 34 (13.5%) had both. Table 1 categorizes the patient cohort based on the type of neurointervention received. Across all groups, the average age ranged from 75.7 to 77.7 years. A higher proportion of patients receiving thrombectomy only were on anticoagulants (33.8%) compared to those receiving tPA only (5%) or both treatments (2.9%). Antiplatelet use was most frequent in patients receiving only tPA (45.7%), with aspirin being the most common. There were 130 patients with missing initial NIHSS scores. All other data was obtained for each patient. All patients underwent initial non-contrast head CT (NCCT) imaging upon presentation. Supplemental diagnostic imaging was performed for the majority of the cohort, as 205 patients (81.7%) received a CT angiogram (CTA) and 200 patients (79.7%) received CT perfusion (CTP) imaging.

**Table 1:** Background characteristics of acute stroke patients presenting to the emergency department by neurointervention received. Data are represented as mean ± standard deviation (SD) for continuous variables (age, blood pressure) and as frequency (N) and percentage (%) for categorical variables (gender, imaging, medication use). tPA: tissue plasminogen activator.

	tPA Only (N=140)	Thrombectomy Only (N=77)	Both (N=34)
Age, years (SD)	76.5 (14.2)	77.7 (12.9)	75.7 (13.2)
Males, n (%)	77 (55.0%)	37 (48.1%)	16 (47.1%)
Systolic BP, mmHg (SD)	154.0 (25.8)	149.9 (26.2)	147.3 (20.1)
Diastolic BP, mmHg (SD)	79.4 (16.9)	82.1 (16.6)	79.2 (18.1)
CT non-contrast, n (%)	140 (100%)	77 (100%)	34 (100%)
CT angiography, n (%)	103 (73.6%)	73 (94.8%)	29 (85.3%)
CT perfusion, n (%)	102 (72.9%)	70 (90.9%)	28 (82.4%)
Any antiplatelet use, n (%)	64 (45.7%)	22 (28.6%)	7 (20.6%)
Single antiplatelet use, n (%)	52 (37.1%)	18 (23.4%)	6 (17.6%)
Dual antiplatelet use, n (%)	12 (8.6%)	4 (5.2%)	1 (2.9%)

Detailed diagnostic findings for these modalities are organized in the following sections. Table 2 presents the results of the initial non-contrast head CT, including the prevalence of acute, subacute, and chronic infarcts. Table 3 summarizes the CT angiography findings, specifically the identification of large vessel occlusions (LVO). Table 4 categorizes the CT perfusion results based on the presence of core and penumbra infarct regions.

**Table 2:** Non-contrast head CT results (n=251)

Finding	Count (n)	Percentage (%)
Normal	200	79.90%
Old infarct	33	13.10%
Acute infarct	16	6.40%
Subacute infarct	2	0.80%
Acute intracerebral hemorrhage (ICH)	1	0.40%
Chronic intracerebral hemorrhage (ICH)	1	0.40%

**Table 3:** CT angiography (CTA) results (n=205)

Finding	Count (n)	Percentage (%)
Large vessel occlusion (LVO)	108	52.70%
Normal	97	47.30%

**Table 4:** CT perfusion (CTP) results (n=200)

Finding	Count (n)	Percentage (%)
Normal	109	54.50%
Both core and penumbra infarcts	44	22.00%
Penumbra infarct	34	17.00%
Core infarct	13	6.50%

Main results

Of the 251 patients included in the study, 42 had ICH after intervention, with a total incidence of 16.7%. The outcomes of the antiplatelet versus no antithrombotic groups are shown in Table 5, while those of anticoagulant versus no antithrombotic groups are shown in Table 6. The incidence of ICH after any neurointervention was 11.9% (95% CI: 4.8 to 19.0), 20.0% (95% CI: 3.1 to 36.9), and 18.0% (95% CI: 11.4 to 24.7), and median mRS was 4 (IQR: 2-4), 4 (IQR: 3-4), and 3 (IQR: 1-4) in the antiplatelet, anticoagulant, and no antithrombotic groups, respectively. Patients in the antiplatelet group who had both tPA and mechanical thrombectomy had a significantly higher mRS versus patients in the no antithrombotic group (5 vs 3; p=0.037). There were no other statistically significant differences in mRS or rate of ICH between the three groups.

**Table 5:** Outcomes of patients by neurointervention group comparing those taking pre-stroke anticoagulants to no antithrombotics. Initial NIHSS (median, IQR) is presented for each clinical subset to provide baseline stroke severity context for that specific intervention group. Each row represents a distinct comparison between patients on the specified antithrombotic regimen and the "No Antithrombotic" control group within that intervention category. Data are represented as median with interquartile range (IQR) for NIHSS and mRS, and as frequency (N) and percentage (%) with 95% confidence intervals (CIs) for subsequent ICH. For all analyses, a p-value of < 0.05 was considered statistically significant. mRS: modified Rankin score, ICH: intracranial hemorrhage, NIHSS: National Institutes of Health Stroke Scale, CH: cerebral hemorrhage, tPA: tissue plasminogen activator.

	Antiplatelet Only	No Antiplatelet	p-Value	OR (95% CI)	Test Statistic
All stroke alert patients with any intervention	N=84	N=133			
Initial NIHSS, median (IQR)	3 (6-13)	6 (3-14)	0.407		U=1119.0
Subsequent ICH, n (%, 95% CI)	10 (11.9%, 4.8 to 19.0)	24 (18.0%, 11.4 to 24.7)	0.225	0.614 (0.277 to 1.359)	χ^2^=1.469
mRS, median (IQR)	4 (2-4)	3 (1-4)	0.093		U=4843.0
tPA only patients (excluding thrombectomy)	N=62	N=71			
Initial NIHSS, median (IQR)	6 (2-10)	5 (3-7)	0.995		U=526.5
Subsequent ICH, n (%, 95% CI)	7 (11.3%, 3.2 to 19.4)	5 (7.0%, 0.9 to 13.1)	0.394	1.680 (0.505 to 5.590)	χ^2^=0.728
mRS, median (IQR)	4 (2-4)	2 (1-4)	0.089		U=1831.5
Thrombectomy only patients (excluding tPA)	N=16	N=35			
Initial NIHSS, median (IQR)	13 (3-14)	10 (6-14.5)	0.820		U=68.0
Subsequent ICH, n (%, 95% CI)	3 (18.8%, -2.7 to 40.2)	12 (34.3%, 17.7 to 50.8)	0.259	0.442 (0.105 to 1.860)	χ^2^=1.277
mRS, median (IQR)	3 (3-4)	3 (1-5)	0.901		U=274.0
Patients with both tPA and thrombectomy	N=6	N=27			
Initial NIHSS, median (IQR)	27 (25-29)	16 (5-26)	0.193		U=3.5
Subsequent ICH, n (%, 95% CI)	0 (0%)	7 (25.9%, 8.3 to 43.6)	0.160	-	χ^2^=1.974
mRS, median (IQR)	5 (4-5)	3 (2-4)	0.037		U=37.5

**Table 6:** Outcomes of patients by neurointervention group comparing those taking pre-stroke antiplatelets to no antithrombotics. Initial NIHSS (median, IQR) is provided for each row to establish the baseline stroke severity of that specific cohort. All outcome comparisons for mRS and ICH rate are made between the medicated group in that row and the intervention-matched 'No Antithrombotic' control group to ensure internal consistency within the analysis. Data are represented as median with interquartile range (IQR) for NIHSS and mRS, and as frequency (N) and percentage (%) with 95% confidence intervals (CIs) for subsequent ICH. For all analyses, a p-value of < 0.05 was considered statistically significant. mRS: modified Rankin score, ICH: intracranial hemorrhage, NIHSS: National Institutes of Health Stroke Scale, CH: cerebral hemorrhage, tPA: tissue plasminogen activator.

	Anticoagulant Only	No Anticoagulant	p-Value	OR (95% CI)	Test Statistic
All stroke alert patients with any intervention	N=25	N=133			
Initial NIHSS, median (IQR)	18 (4-26)	6 (3-14)	0.055		U=276.5
Subsequent ICH, n (%, 95% CI)	5 (20.0%, 3.1 to 36.9)	24 (18.0%, 11.4 to 24.7)	0.817	1.135 (0.388 to 3.327)	χ^2^=0.054
mRS, median (IQR)	4 (3-4)	3 (1-4)	0.246		U=1423.0
tPA only patients (excluding thrombectomy)	N=5	N=71			
Initial NIHSS, median (IQR)	13.5 (1-26)	5 (3-7)	1.000		U=34.0
Subsequent ICH, n (%, 95% CI)	0 (0%)	5 (7.0%, 0.9 to 13.1)	0.539	-	χ^2^=0.377
mRS, median (IQR)	1 (1-3)	2 (1-4)	0.563		U=150.5
Thrombectomy only patients (excluding tPA)	N=20	N=35			
Initial NIHSS, median (IQR)	18 (6-25.5)	10 (6-14.5)	0.178		U=67.0
Subsequent ICH, n (%, 95% CI)	5 (25.0%, 4.2 to 45.8)	12 (34.3%, 17.7 to 50.8)	0.473	0.639 (0.187 to 2.185)	χ^2^=0.514
mRS, median (IQR)	4 (3-5)	3 (1-5)	0.479		U=310.5
Patients with both tPA and thrombectomy	N=0	N=27			
Initial NIHSS, median (IQR)		16 (5-26)	-		-
Subsequent ICH, n (%, 95% CI)		7 (25.9%, 8.3 to 43.6)	-	-	-
mRS, median (IQR)		3 (2-4)	-		-

Patients in the combined anticoagulant and antiplatelet group had an ICH incidence of 33.3% (95% CI: 5.1 to 71.8), and a median mRS of 5 (IQR: 4-5). These patients had a significantly higher mRS versus patients in the unmedicated group (5 vs 3; p=0.033). There were no other statistically significant differences in mRS or incidence of ICH between groups (Table 7). Additionally, there were no statistically significant differences in mRS or rate of ICH in comparing the subgroups of single versus dual antiplatelet usage (Table 8).

**Table 7:** Outcomes of patients by neurointervention group comparing those taking both anticoagulants and antiplatelets pre-stroke to no antithrombotics. Data are represented as median with interquartile range (IQR) for NIHSS and mRS, and as frequency (N) and percentage (%) with 95% confidence intervals (CIs) for subsequent ICH. For all analyses, a p-value of < 0.05 was considered statistically significant. mRS: modified Rankin score, ICH: intracranial hemorrhage, AC: anticoagulant, AP: antiplatelet, NIHSS: National Institutes of Health Stroke Scale, CH: cerebral hemorrhage, tPA: tissue plasminogen activator.

	Both AC and AP	Neither	p-Value	OR (95% CI)	Test Statistic
All stroke alert patients with any intervention	N=9	N=133			
Initial NIHSS, median (IQR)	15 (11-22)	6 (3-14)	0.152		U=114.0
Subsequent ICH, n (%, 95% CI)	3 (33.3%, -5.1 to 71.8)	24 (18.0%, 11.4 to 24.7)	0.258	2.271 (0.530 to 9.726)	χ^2^=1.279
mRS, median (IQR)	5 (4-5)	3 (1-4)	0.033		U=349.0
tPA only patients (excluding thrombectomy)	N=2	N=71			
Initial NIHSS, median (IQR)	8 (1-15)	5 (3-7)	0.780		U=30.0
Subsequent ICH, n (%, 95% CI)	0 (0%)	5 (7.0%, 0.9 to 13.1)	0.697	-	χ^2^=0.151
mRS, median (IQR)	3 (1-4)	2 (1-4)	0.863		U=66.0
Thrombectomy only patients (excluding tPA)	N=6	N=35			
Initial NIHSS, median (IQR)	22 (15-26)	10 (6-14.5)	0.064		U=7.5
Subsequent ICH, n (%, 95% CI)	2 (33.3%, -20.9 to 87.5)	12 (34.3%, 17.7 to 50.8)	0.964	0.958 (0.153 to 6.006)	χ^2^=0.002
mRS, median (IQR)	5 (4-5)	3 (1-5)	0.087		U=58.0
Patients with both tPA and thrombectomy	N=1	N=27			
Initial NIHSS, median (IQR)	11 (11-11)	16 (5-26)	0.600		U=3.0
Subsequent ICH, n (%, 95% CI)	1 (100%)	7 (25.9%, 8.3 to 43.6)	0.107	-	χ^2^=2.593
mRS, median (IQR)	5 (5-5)	3 (2-4)	0.181		U=3.0

**Table 8:** Outcomes of patients by neurointervention group comparing those taking pre-stroke single antiplatelet to dual antiplatelet, excluding patients taking anticoagulants. Data are represented as median with interquartile range (IQR) for NIHSS and mRS, and as frequency (N) and percentage (%) with 95% confidence intervals (CIs) for subsequent ICH. For all analyses, a p-value of < 0.05 was considered statistically significant. mRS: modified Rankin score, ICH: intracranial hemorrhage, AP: antiplatelet, NIHSS: National Institutes of Health Stroke Scale, CH: cerebral hemorrhage, tPA: tissue plasminogen activator.

	Dual AP	Single AP	p-Value	OR (95% CI)	Test Statistic
All stroke alert patients with any intervention	N=17	N=67			
Initial NIHSS, median (IQR)	6.5 (3-10)	6 (2.5-13)	0.871		U=103.5
Subsequent ICH, n (%, 95% CI)	1 (5.9%, -6.6 to 18.4)	9 (13.4%, 5.1 to 21.8)	0.391	0.403 (0.047 to 3.419)	χ^2^=0.737
mRS, median (IQR)	4 (3-4)	4 (2-4)	0.875		U=477.0
tPA only patients (excluding thrombectomy)	N=12	N=50			
Initial NIHSS, median (IQR)	4 (3-9)	6 (2-11)	0.705		U=58.0
Subsequent ICH, n (%, 95% CI)	1 (8.3%, -10.0 to 26.7)	6 (12.0%, 2.7 to 21.3)	0.719	0.667 (0.073 to 6.124)	χ^2^=0.130
mRS, median (IQR)	4 (3-5)	4 (2-4)	0.149		U=221.0
Thrombectomy only patients (excluding tPA)	N=4	N=12			
Initial NIHSS, median (IQR)	14 (14-14)	9 (2-16)	0.667		U=2.5
Subsequent ICH, n (%, 95% CI)	0 (0%)	3 (25.0%, -3.7 to 53.7)	0.267	-	χ^2^=1.231
mRS, median (IQR)	4 (4-4)	3 (2-4)	0.212		U=13.5
Patients with both tPA and thrombectomy	N=1	N=5			
Initial NIHSS, median (IQR)	-	27 (25-29)	-		-
Subsequent ICH, n (%, 95% CI)	0 (0%)	0 (0%)	-	-	-
mRS, median (IQR)	2 (2-2)	5 (5-5)	0.120		U=0.0

## Discussion

This retrospective review analyzed post-intervention intracranial hemorrhage and modified Rankin score in patients who presented to a southeast Florida stroke center. The ICH rate in this study was consistent with rates found in previous literature [8]. Among patients who received both thrombolytics and neurointervention, patients taking pre-stroke antiplatelet agents alone or in combination with an anticoagulant agent had a higher mRS score than unmedicated patients. These findings were not accompanied by changes in the rate of ICH. As this study is not a randomized controlled trial, it is difficult to conclude that the difference in the disability scale is due to the presence of an antithrombotic at the time of neurointervention. A plausible explanation is that the comorbidities requiring these medications adversely affected patient outcomes. Prior studies have also shown that having comorbidities, such as atrial fibrillation, have a negative effect on outcomes [9]. Furthermore, the correlation between antithrombotic use and initial stroke severity, evidenced by the higher median NIHSS in the anticoagulant group (18 vs. 6; p=0.055) in our cohort, suggests that medicated patients may have had a more severe initial presentation. This supports the conclusion that their functional recovery (mRS) was influenced by their baseline clinical state and known comorbid conditions rather than the neurointerventional treatment itself.

There was no significant difference in the rate of ICH between any of the three groups after any neurointervention. In this dataset, no increased ICH was observed, suggesting that pre-stroke antithrombotic medication use may not preclude neurointervention treatments; however, these findings remain hypothesis-generating. In addition, other studies have shown similar findings [10-16]. The 2026 AHA/ASA Guidelines have recently moved toward tenecteplase (TNK) as a preferred thrombolytic agent for its ease of administration and fibrin specificity (Class 1 recommendation). While these guidelines establish safety for antiplatelet users, they designate the benefit-risk ratio for patients on direct oral anticoagulants (DOACs) as uncertain [4]. A 2022 retrospective analysis of DOAC patients receiving alteplase for acute ischemic stroke did not find an association with intracranial hemorrhage [10]. A similar study of patients on a DOAC with an acute stroke that received tPA also found no significant difference in ICH [11]. It is important to note that our reported ICH rates encompass all radiographic evidence of hemorrhage. In many cases, post-reperfusion hemorrhage is asymptomatic and does not necessarily preclude a favorable long-term functional outcome. Because our study did not distinguish between symptomatic and asymptomatic ICH, our safety findings may be viewed as a conservative estimate of the risk associated with pre-stroke antithrombotic use.

In contrast to the uncertainty surrounding chemical thrombolysis, mechanical thrombectomy is increasingly viewed as a safe primary intervention for anticoagulated patients. In a retrospective analysis of 94 patients receiving mechanical thrombectomy, there was no increased risk of ICH in patients on therapeutic anticoagulation [15]. A larger retrospective study of patients receiving mechanical thrombectomy found that ICH was not associated with anticoagulant or antiplatelet use [12]. Studies of patients on warfarin treated with TPA also suggest that ICH rates are not increased [16-18].

The results of our study and the studies cited above suggest that there may be a role for endovascular treatments for patients on anticoagulant or antiplatelet agents that present with an acute stroke. Given the "uncertainty" highlighted in the 2026 guidelines regarding chemical thrombolysis in medicated patients, our findings provide important real-world evidence for clinicians performing this risk-benefit calculus [4]. These data justify the performance of prospective randomized controlled studies. Thrombolytics and thrombectomy offer improved outcomes in acute stroke. A substantial portion of patients with an acute stroke are being treated with antiplatelet or anticoagulant agents and may benefit from these emergency neurointerventional treatments. Further study should provide additional information to help inform this critical risk-benefit analysis.

Limitations

As a retrospective study, the duration of follow-up exhibited considerable variation among patients. Some individuals had neuroimaging and neurological condition documentation extending many months after the intervention, while others had records only up until their discharge, which in some cases was as brief as two days. Additionally, patients who experienced ICH after discharge and subsequently sought treatment at another medical facility might have been lost to follow-up, although these patients should have been transferred back to the study hospital if this had occurred.

Several critical clinical variables were not captured in this review, which may influence the interpretation of functional outcomes. These include stroke etiology (e.g., cardioembolic vs. large-artery atherosclerosis) and baseline imaging scores such as Alberta Stroke Program Early CT Score (ASPECTS) or pc-ASPECTS. Furthermore, lifestyle factors such as significant alcohol use, cigarette smoking, and electronic cigarette vaping were not accounted for, nor was pre-morbid clinical frailty.

The initial documentation of the NIHSS score was not consistent across all charts included in the study. Many different physicians and associated providers authored these charts, resulting in inconsistent documentation of the NIHSS score. In our study hospital, NIHSS scores were initially recorded on paper by nursing staff but may not have been entered into the EMR. Missing NIHSS data would be expected to be uniform across all patient groups, and this variable was not used in the primary analysis.

The review of each patient's medication list was performed by a combination of internal medicine staff, multiple consultants, and nursing personnel. While the patients' home medication lists were available, information about the timing of their last doses of antiplatelet or anticoagulant medications prior to neurointervention was often not known or was found to be inaccurate. Further, patient compliance to prescribed medication usage was not always known. This potential lack of accuracy in medication data could introduce biases in data analysis.

In our study, eight patients who were taking an anticoagulant medication received tPA. While therapeutic anticoagulation is a historical contraindication, the 2026 guidelines suggest the risk-benefit profile is uncertain in some contexts. However, the specific medical decision-making behind these "off-label" treatments remains unknown in this retrospective context. It is possible the patients’ anticoagulant use was uncertain at the tPA administration but later became known. Alternatively, the treating physician might have been comfortable treating the patient with tPA anyway. In addition, the sample size was relatively small, as our study only included 251 patients. The absence of statistical significance in certain comparisons may be attributed to inadequate statistical power rather than a true absence of differences between groups.

## Conclusions

Acute stroke patients using daily anticoagulant or antiplatelet agents do not appear to have increased ICH risk when treated with thrombolytics or thrombectomy. Anticoagulant or antiplatelet use may not be a valid contraindication for acute stroke neurointerventions. Randomized controlled trials studying this issue are warranted.
